# Hand grip strength and plasma markers of neurodegeneration among 1026 community‐dwelling older Nigerian adults: Insights from the VALIANT Study

**DOI:** 10.1002/alz70856_104142

**Published:** 2025-12-26

**Authors:** Oladotun V Olalusi, Tolulope O Akinyemi, Andrea L. Benedet, Gabriel O Ogunde, Joseph O Yaria, Eniola O Cadmus, Femi O Popoola, Mayowa Ogunronbi, Dorcas Olujobi, Olaoluwa Famuyiwa, Joshua O Akinyemi, Mayowa O Owolabi, Roman Romero‐Ortuno, Chinedu T Udeh‐Momoh, Adesola Ogunniyi, Margaret Pericak‐Vance, Raj Kalaria, Brian Lawlor, Henrik Zetterberg, Rufus O. Akinyemi

**Affiliations:** ^1^ Neuroscience and ageing research unit, Institute of Advanced Medical Research and Training (IAMRAT), College of Medicine, University of Ibadan, Ibadan Nigeria, Oyo, Nigeria; ^2^ Department of Neurology, University College Hospital, Ibadan, Oyo, Nigeria; ^3^ John P. Hussman Institute for Human Genomics, University of Miami, Miami, FL, USA; ^4^ Lead City University, Ibadan, Oyo, Nigeria; ^5^ Department of Psychiatry and Neurochemistry, Institute of Neuroscience and Physiology, The Sahlgrenska Academy, University of Gothenburg, Mölndal, Sweden; ^6^ Institute for Advanced Medical Research and Training, College of Medicine, University of Ibadan, Ibadan, Oyo, Nigeria; ^7^ University College Hospital, Ibadan, Oyo, Nigeria; ^8^ University of Ibadan and University College Hospital, Ibadan, Oyo, Nigeria; ^9^ University of Ibadan College of Medicine, Ibadan, Oyo, Nigeria; ^10^ College of Medicine, University of Ibadan, Ibadan, Oyo, Nigeria; ^11^ Institute of Advanced Medical Research and Training (IAMRAT), College of Medicine, University of Ibadan, Ibadan Nigeria, Oyo, Nigeria; ^12^ Neuroscience And Ageing Research Unit, Institute for Advanced Medical Research and Training, College of Medicine, University of Ibadan, Ibadan, Nigeria; ^13^ College of Medicine, University of Ibadan, Ibadan, Oyo State, Nigeria; ^14^ Global Brain Health Institute, Trinity College, Dublin, Ireland; ^15^ Wake Forest University School of Medicine, Winston‐Salem, NC, USA; ^16^ Brain and Mind Institute, Aga Khan University, Nairobi Province, Nairobi, Kenya; ^17^ Institute for Advanced Medical Research and Training, College of Medicine, University of Ibadan, Ibadan, Nigeria; ^18^ Africa Dementia Consortium, Ibadan, Ibadan, Nigeria; ^19^ John P. Hussman Institute for Human Genomics, University of Miami Miller School of Medicine, Miami, FL, USA; ^20^ Newcastle University, Translational and Clinical Research Institutetitute for Ageing and Health, Newcastle UniversityNewcastle University, Newcastle upon Tyne, United Kingdom; ^21^ Global Brain Health Institute, Trinity College Dublin, Dublin, Ireland; ^22^ Trinity College Institute of Neuroscience, School of Psychology, Trinity College Dublin, Dublin, Ireland; ^23^ St James Hospital, Dublin, Ireland; ^24^ Department of Psychiatry and Neurochemistry, Institute of Neuroscience and Physiology, the Sahlgrenska Academy, University of Gothenburg, Molndal, Sweden; ^25^ Department of Psychiatry and Neurochemistry, Institute of Neuroscience and Physiology, The Sahlgrenska Academy at the University of Gothenburg, Mölndal, Västra Götalands län, Sweden; ^26^ University College London, London, United Kingdom, London, United Kingdom; ^27^ Institute of Advanced Medical Research and Training, College of Medicine, University of Ibadan, Ibadan, Oyo State, Nigeria

## Abstract

**Background:**

Plasma biomarkers as well as non‐invasive measures of frailty are increasingly employed to aid population‐wide cognitive screening, phenotyping and preclinical diagnosis. Hand grip strength (HGS), a measure of physical and cardiometabolic health, is indicative of early cognitive decline but its relationship with plasma biomarkers of Alzheimer's Disease (AD) and neurodegeneration is not known. We investigated the association between HGS and plasma markers of AD and neurodegeneration in the Vascular heAlth, fraiLty, and cognItion in Ageing Nigerians (VALIANT) study.

**Method:**

**VALIANT** is a longitudinal community‐based cohort study. One thousand and thirty‐six (1036) participants were recruited from Wards 2 and 3 of the Ibadan Northeast local government area of Oyo State, Nigeria through a multi‐stage, stratified cluster random sampling method. Initial screening for cognitive dysfunction was performed using the IDEA, and MoCA scores. Patients were then diagnosed as having dementia and MCI via a consensus diagnosis of at least two neurologists. Glial fibrillary acidic protein (GFAP), neurofilament light chain (NfL), phosphorylated tau (*p*‐tau)217 and amyloid beta (Aβ)42/40 were measured using ultra‐sensitive immunoassays. Biomarker data were log‐ and square‐root transformed. Multiple linear regression analysis was employed to detect the relationship between HGS and plasma biomarkers, while adjusting for sociodemographic, lifestyle, cardiometabolic, social and cognitive function scores. β coefficient and corresponding 95% CI was reported. (*p*‐value <0.05)

**Result:**

At baseline, a total of 35 participants (3.4%) and 112 (11%) had dementia and mild cognitive impairment (MCI) respectively. The mean (SD) age of participants was 77.0 (11.3) dementia, 72.6 (9.8) MCI and 63.8 (9.9) normal (*p* value<0.001). In the simple linear regression, significant bivariate relationship was observed between HGS and all the plasma biomarkers. In the multiple linear regression analysis, HGS showed an independent relationship with respective β (95% CI) of ‐0.0026 (‐0.0051, ‐0.0001) for pTau217, 0.009 (0.002, 0.016) for Ab42, and ‐0.013 (‐0.021, ‐0.004) for NfL.

**Conclusion:**

Hand grip strength is independently associated with plasma markers of neurodegeneration in this cohort of community‐dwelling older Nigerians individuals. Both measures could serve as potential screening tools for identifying individuals at risk for AD/ADRD, complementing other existing biomarkers.

